# Decreased expression of eukaryotic initiation factor 3f is an adverse prognostic factor for stage I–III gastric cancer

**DOI:** 10.1186/1477-7819-12-72

**Published:** 2014-03-28

**Authors:** Guanghua Li, Na Wang, Chuanjin Sun, Bo Li

**Affiliations:** 1 Department of Oncology, The People’s Hospital of Zhangqiu, Shandong Province, 38th Huiquan Road, Jinan 250200, China; 2 Department of Nephrology, The People’s Hospital of Zhangqiu, Shandong Province, 38th Huiquan Road, Jinan 250200, China; 3 Central Electrocardiogram Department, The People’s Hospital of Zhangqiu, Shandong Province, 38th Huiquan Road, Jinan 250200, China; 4 Department of General Surgery, The Second Hospital of Shandong University, 247th Beiyuan Avenue, Jinan 250200, China

**Keywords:** Biomarker, eIF3f, Gastric cancer, Prognosis

## Abstract

**Background:**

It has been demonstrated that eIF3f expression is significantly decreased in many human cancers, a fact which plays an important role in human cancer. However, the expression of eIF3f in gastric cancer (GC) is not well understood to date. Therefore, the aim of this study is to detect the expression of eIF3f in GC.

**Methods:**

The expression of eIF3f was examined by immunohistochemistry in tissues with stage I to III GC and adjacent non-cancerous tissues (ANCT) of 195 gastrectomy specimens; clinicopathological results, including survival, were analyzed.

**Results:**

The positive expression rate of eIF3f was significantly higher in ANCT tissues than in GC. eIF3f levels were correlated with more advanced tumor stages and likelihood of recurrence (all *P* <0.05). The Kaplan-Meier survival curves indicated that decreased expression of eIF3f could serve as a prognosis marker for poor outcome of GC patients (*P* = 0.04).

**Conclusions:**

eIF3f may play an important role in recurrence, thus representing a promising predictive marker for the prognosis of GC.

## Background

From a global perspective, gastric cancer (GC) is the fourth most common malignant tumor and the second cause of cancer-related death, accounting for an estimated 989,600 new cases and 738,000 deaths in 2008 [1-3]. In the past several decades, intensive efforts have been made to identify tools to improve prognostication of gastric cancer; miR-21, miRNA-106a, and miR-143, as well as miR-203 are considered to be promising novel biomarkers for GC [4]. However, except for the cancer staging, upon which adjuvant treatment depends, definite predictive factors for the prognosis of GC are relatively scarce [5-8]. Thus, finding new prognostic tools, such as biomarkers, for the accurate diagnosis of GC is of vital importance to improve the therapeutic effect and prolong the survival of patients.

The eukaryotic initiation factor 3f (eIF3f) is the p47 subunit of the multi-subunit eIF3 complex and plays an important role in translation initiation [9-11]. Although the role of eIF3f in the eIF3 complex has not been defined [12-14], shut-off experiments in Schizosaccharomyces pombe showed that in a long-term period, eIF3f was essential for viability [15]. The translation system indicated that, in mammalian cells, eIF3f was a negative regulator of translation, which played an important role in human cancer [12,16]. A study by Marchione et al. showed that the f subunit of eIF3 represented a promising candidate molecule to use for bio-therapeutic applications [17]. Previous studies have identified eIF3f as a protein involved in apoptotic signaling as a negative regulator of translation and have also demonstrated that eIF3f expression significantly decreased in many human cancers, and thus played an important role in human cancer [11,16,18,19]. Shi et al. reported that eIF3f was downregulated in several human tumors, and that its overexpression inhibited cell proliferation in melanoma and pancreatic cancer cells [11]. A recent study by Higareda-Mendoza and Pardo-Galván also showed that eIF3f was essential for A549 cell proliferation and its absence induced the cell to enter apoptosis [20].

Together, these previous findings suggest that eIF3f may be involved in the regulation of cell growth and proliferation and contributes to tumorigenesis; however, its role in GC remains unclear. Thus, in order to shed light into the expression of eIF3f in GC, we correlated eIF3f expression results with the clinicopathologic characteristics and prognosis of GC patients. We conducted experiments to characterize the localization of eIF3f by immunohistochemistry, aiming to find a new prognostic marker for GC and new diagnosis and treatment strategies.

## Methods

### Patient samples

A review of surgical specimens at the The Second Hospital of Shandong University between November 2007 and March 2009 yielded 195 cases of GC patients who had undergone curative surgery. The mean age of the patients was 62 years (range, 31–90 years). The median follow-up period was 38 months (range: 3–54 months). The TNM stage of tumors was assessed according to the 7th ed. of the TNM Classification of GC [21]. The Her-2/neu overexpression was detected by postoperative fluorescence in situ hybridization examination. No patient underwent radiotherapy or chemotherapy before operation.

For immunohistochemical staining, the paraffin-embedded tissue sections were obtained from 195 cases of radical resection of GC. The fragments were fixed in neutral formol. All the cases were reviewed by a pathologist to confirm the malignancy. The investigation was approved by the ethics committee of the medical faculty and written informed consents were obtained from all patients.

### Immunohistochemistry

The polyclonal antibody (ab64177) to eIF3f was bought from Abcom (Cambridge, UK). The paraffin-embedded tissue blocks were sectioned in 3 to 4 mm slices and placed on Anti slides. After de-waxing and hydration, the slides were rinsed in phosphate-buffered saline (PBS) and blocked for 10 min with 3% hydrogen peroxide to deprive the endogenous peroxidase activity. After antigen retrieval with the use of a microwave, the specimens were incubated with the anti eIF3f MAb (diluted 1:100 in PBS) at 37°C for 1.5 hours. The sections were then washed 3 × 3 min in PBS and incubated with 1 and 2 Reagent of PV9000 Mouse/Rabbit hypersensitivity two-step immunohistochemical Kit (Beijing fir Jinqiao, Beijing, China) for a total of 60 min at 37°C in a humid chamber. The sections were washed 3 × 3 min with PBS, followed by the addition of diaminobenzidine as a chromogen for 3 to 5 min, which was strictly controlled under a microscope. Antibodies were optimized using a positive control tissue according to the manufacturer’s instructions. In negative controls, the primary antibody was replaced with PBS. The remaining procedures were performed in parallel with other specimens. Each slide was scored in a blinded fashion by two pathologists according to the manufacturer’s recommended criteria at × 100 and × 200 magnification. The overall percentage of positive cells on an immunostained section was determined according to the pattern of intracellular localization. The extent and pattern of the eIF3f-specific immunostaining within a tissue section were determined by the percentage of cells with cytoplasm staining. The immunostaining was read in a semiquantitative manner. Three visual fields were examined randomly and the rate of positive cells was divided into less than 5% (score 0), 6% to 25% (score 1), 26% to 50% (score 2), 51% to 75% (score 3), and more than 75% (score 4). The staining intensity can be divided into three grades: no staining (score 0), slightly yellowish (score 1), brownish yellow (score 2), and dark brown (score 3). The multiplication of the two were graded as follows: 0 (score 0), 1+ (score 1–4), 2+ (score 5–8), and 3+ (score 9–12). Intensity scores of 0 or 1+ were designated as low expression and 2+ or 3+ were designated as high expression.

### Statistical analysis

Differences between groups were analyzed using a Student’s *t*-test for continuous variables and a χ^2^ test or Fisher’s exact test for proportions. The overall survival rate was estimated by the Kaplan-Meier method. Univariate analyses (Cox proportional hazard regression models) were also performed to assess the prognostic value of nucleolin expression and other clinicopathological features. We utilized SAS 9.2 software system for statistical analysis.

## Results

### eIF3f expression and clinicopathological characteristics

From the 195 cases, 142 men and 53 women all accepted radical operation of GC, including 116 primary and 79 recurrent GC patients. Pathologically, all of the 195 cases were adenocarcinoma, which covered 12 well differentiated, 27 moderate differentiated, 156 low differentiated, and 4 signet-ring cell carcinoma. For all specimens, 31 cases were demonstrated as Her-2/neu-positive.

### eIF3f expression in gastric cancer and ANCT

**Figure 1:**
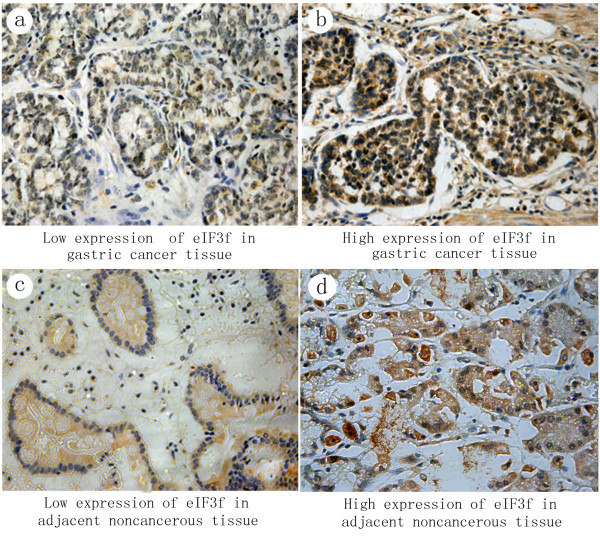
**Immunohistochemical findings of the degree of expressions of eIF3f in gastric cancer (GC) tissue and adjacent non-cancerous tissue (ANCT).** eIF3f was localized in the cytoplasm at different levels and percentages. **(a)** and **(b)** show eIF3f staining in GC tissue: low expression **(a)** and high expression **(b)** (magnification × 400). **(c)** and **(d)** show eIF3f staining in ANCT: low expression **(c)** and high expression **(d)** (magnification × 400).

Immunohistochemical staining for eIF3f showed a cytoplasm staining pattern in both GC (Figure 1a, b) and ANCT (Figure 1c, d) tissues. Immunoscores were calculated when the presence of eIF3f deposits in tumor glands and cytoplasmic staining were positive. The rate of high eIF3f expression in GC and ANCT tissues was 33.8% (66/195) and 59.5% (116/195), respectively.

### Correlation between eIF3f expression and clinicopathological features

The association of eIF3f levels with clinicopathological features in GC was also assessed in Table 1. Low eIF3f expression in GC was significantly associated with more advanced tumor stages (*P* = 0.02) and likelihood of recurrence (*P* = 0.04). However, eIF3f expression was not significantly correlated with sex (*P* = 0.87), age (*P* = 0.17), tumor differentiation (*P* = 0.46), and tumor size (*P* = 0.16). Out of 136 stage I–II cases, 53 (40.0%) had high eIF3f staining densitometry values, and these were higher than cases of stage III (*P* = 0.02). Furthermore, the overall densitometric analysis of immunostaining revealed that the downregulated eIF3f protein expression varied significantly between primary and recurrent tumors (*P* = 0.04).

**Table 1:** Correlation among eIF3f staining and clinical characteristics

**Characteristics**	**No.**	**eIF3f-high (n = 66)**	**eIF3f-low (n = 129)**	***P***
Gender				
Male	142	49	93	0.87
Female	53	17	36	
Age (y)				
<60	84	33	51	0.17
≥60	111	33	78	
Tumor differentiation				
Well	12	8	4	0.46
Moderate	27	4	23	
Poor	156	54	102	
Tumor size (cm)				
<4.5	114	34	80	0.16
≥4.5	81	32	49	
TNM stage				
I–II	136	53	83	0.02
III	59	13	46	
Her-2 status				
Negative	164	53	111	0.30
Positive	31	13	18	
Primary/Recurrent				
Primary tumor	116	46	70	0.04
Recurrent tumor	79	20	59	

### The relationship between the expression level of eIF3f and prognosis

**Figure 2:**
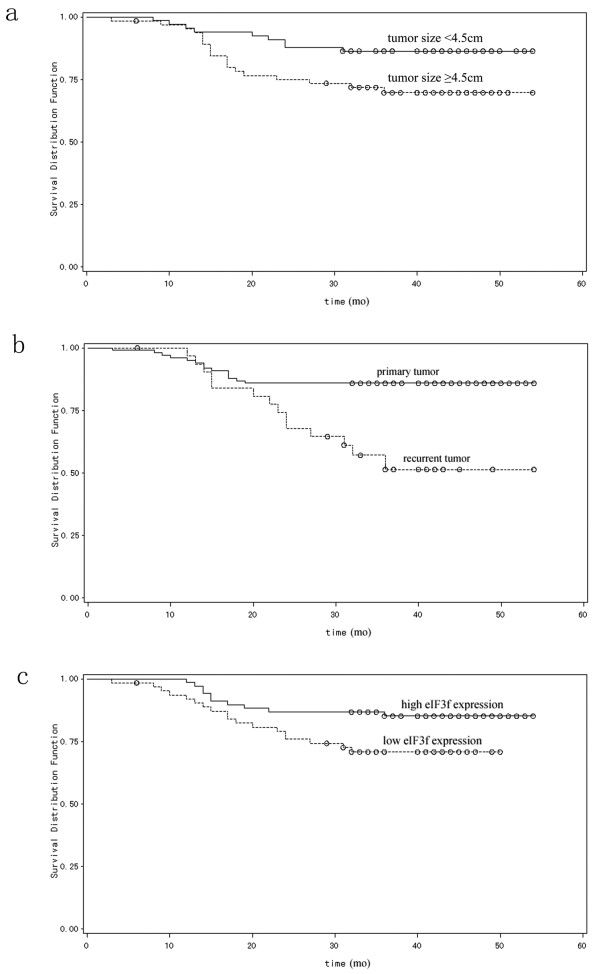
**Overall survival curves for gastric cancer (GC) patients. (a)** Kaplan-Meier overall survival curves for GC patients with tumor size <4.5 cm and ≥4.5 cm. GC patients with tumor size ≥4.5 cm had significantly shorter overall survival (*P* = 0.03) than those <4.5 cm. **(b)** Kaplan-Meier overall survival curves for primary or recurrent GC patients. Recurrent GC patients had significantly shorter overall survival (*P* <0.01) than primary GC patients. **(c)**. A comparison of the overall survival curve between patients with low and high eIF3f expression. The median progression-free survival was 42.5 months in patients with high eIF3f expression, compared to 33 months in patients with low eIF3f expression.

The prognostic effect of eIF3f on the survival rate of GC patients was investigated by comparing the survival rate of patients with high or low levels of eIF3f protein expression in tumors using Kaplan-Meier survival curves and log-rank test. Univariate Cox regression analysis showed that clinical variables, including tumor size (log-rank, *P* = 0.03; Figure 2a), tumor recurrence (log-rank, *P* <0.01; Figure 2b), and eIF3f expression (log-rank, *P* = 0.04; Figure 2c) were all significantly associated with overall survival (Table 2). Data indicates that high expression of eIF3f protein was a significant prognostic factor for better survival of GC patients. Furthermore, multivariate Cox regression analyses showed that eIF3f expression was an independent predictor for the overall survival of patients with GC.

**Table 2:** Univariate and multivariate analysis of overall survival in patients with gastric cancer

		**No.**	**Hazard ratio**	***P***
			**(95% CI)**	
Univariate analysis				
Gender				
	Male	142		0.40
	Female	53		
Age (y)				
	<60	84		0.12
	≥60	111		
Tumor differentiation				
	Well	12		0.74
	Moderate	27		
	Poor	156		
Tumor size (cm)				
	<4.5	114		0.03
	≥4.5	81		
TNM stage				
	I–II	136		0.88
	III	59		
Her-2 status				
	Negative	164		0.12
	Positive	31		
Primary/Recurrent				
	Primary tumor	116		<0.01
	Recurrent tumor	79		
eIF3f expression				
	Low	129		0.04
	High	66		
Multivariate analysis				
Tumor size (cm)				
	<4.5	114	2.31	0.04
	≥4.5	81	1.04–5.11	
Primary/Recurrent				
	Primary tumor	116	3.77	<0.01
	Recurrent tumor	79	1.79–7.94	
eIF3f expression				
	Low	129	2.27	0.04
	High	66	1.05–4.92	

## Discussion

Although the incidence and mortality rate of GC have fallen over the past several decades, GC is still the fourth most common cancer and the second leading cause of cancer-related death in the world [22,23]. Disappointingly, little improvement has been achieved within the past several decades despite advances in tumor diagnosis and treatment [24,25]. Given the high failure rate of conventional treatment strategies, many cancer-related molecules have been characterized with the goal of developing novel anticancer strategies.

eIF3f is another eIF3 subunit whose function is not well-known [18,26]. Previous studies have demonstrated that overexpression of eIF3f inhibits cell proliferation and induces apoptosis in melanoma and pancreatic cancer cells, suggesting that downregulation of eIF3f is involved in tumorigenesis for many types of cancer [11,16,19]. However, there is no large sample report about the expression and clinical significance of eIF3f in human GC progression and prognosis. In this study, we investigated the expression of eIF3f in GC as well as its correlation with the clinicopathological features and prognosis.

The data from our study showed that the levels of eIF3f protein were significantly lower in GC tissue compared with paired ANCT. Additionally, the decreased expression of eIF3f protein was significantly related to more advanced tumor stages (*P* = 0.02) and likelihood of recurrence (*P* = 0.04). No statistically significant relationship was found between the eIF3f levels and sex (*P* = 0.87), age (*P* = 0.17), tumor differentiation (*P* = 0.46), and tumor size (*P* = 0.16). The Kaplan-Meier survival curves showed that patients with low eIF3f expression had significantly poorer survival compared with patients with high eIF3f expression. Furthermore, multivariate Cox regression analyses showed that tumor size (*P* = 0.04), recurrence (*P* <0.01), and eIF3f expression (*P* = 0.04) were all prognostic predictors of human GC after resection. Based on these results, we propose a bold hypothesis that a preoperative determination of eIF3f expression may be useful in predicting the therapeutic effect and postoperative survival of human GC. A drawback of the present study is the lack of investigation of eIF3f at the molecular and cellular levels in GC. Therefore, further studies should be performed for a more concrete evidence to support the correlation between eIF3f expression and the malignant degree of GC.

## Conclusion

In summary, eIF3f is downregulated in GC. Ultimately, eIF3f may play an important role in the progression and recurrence of GC. However, the specific regulatory mechanisms remain to be further studied to provide potential targets for the treatment of GC.

## Abbreviations

ANCT: Adjacent non-cancerous tissues; eIF3f: Eukaryotic initiation factor 3f; GC: Gastric carcinoma; PBS: Phosphate-buffered saline; TNM: Tumor, node, metastasis.

## Competing interests

The authors declare that they have no competing interest.

## Authors’ contributions

GL designed the study and gave conceptual advice; CS performed experiments; BL collected data and performed the follow-up work of all patients; NW analyzed data and wrote the manuscript. All authors read and approved the final manuscript.
